# Fabrication of a reticular poly(lactide-co-glycolide) cylindrical scaffold for the *in vitro* development of microvascular networks

**DOI:** 10.1080/14686996.2016.1278351

**Published:** 2017-03-01

**Authors:** Yen-Ting Tung, Cheng-Chung Chang, Jyh-Cherng Ju, Gou-Jen Wang

**Affiliations:** ^a^ Program in Tissue Engineering and Regenerative Medicine, National Chung-Hsing University, Taichung, Taiwan; ^b^ Graduate Institute of Biomedical Engineering, National Chung Hsing University, Taichung, Taiwan; ^c^ Department of Mechanical Engineering, National Chung Hsing University, Taichung, Taiwan; ^d^ Core laboratory for Stem Cell Research, Medical Research Department, China Medical University Hospital, Taichung, Taiwan; ^e^ Department of Animal Science, National Chung Hsing University, Taichung, Taiwan; ^f^ Department of Bioinformatics and Medical Engineering, Asia University, Taichung, Taiwan

**Keywords:** Microvascular network, cylinder PLGA scaffold, human umbilical vein endothelial cell, 30 Bio-inspired and biomedical materials, 211 Scaffold / Tissue engineering/Drug delivery

## Abstract

The microvascular network is a simple but critical system that is responsible for a range of important biological mechanisms in the bodies of all animals. The ability to generate a functional microvessel not only makes it possible to engineer vital tissue of considerable size but also serves as a platform for biomedical studies. However, most of the current methods for generating microvessel networks *in vitro* use rectangular channels which cannot represent real vessels *in vivo* and have dead zones at their corners, hence hindering the circulation of culture medium. We propose a scaffold-wrapping method which enables fabrication of a customized microvascular network *in vitro* in a more biomimetic way. By integrating microelectromechanical techniques with thermal reflow, we designed and fabricated a microscale hemi-cylindrical photoresist template. A replica mold of polydimethylsiloxane, produced by casting, was then used to generate cylindrical scaffolds with biodegradable poly(lactide-co-glycolide) (PLGA). Human umbilical vein endothelial cells were seeded on both sides of the PLGA scaffold and cultured using a traditional approach. The expression of endothelial cell marker CD31 and intercellular junction vascular endothelial cadherin on the cultured cell demonstrated the potential of generating a microvascular network with a degradable cylindrical scaffold. Our method allows cells to be cultured on a scaffold using a conventional culture approach and monitors cell conditions continuously. We hope our cell-covered scaffold can serve as a framework for building large tissues or can be used as the core of a vascular chip for *in vitro* circulation studies.

## Graphical abstract

**Figure:**
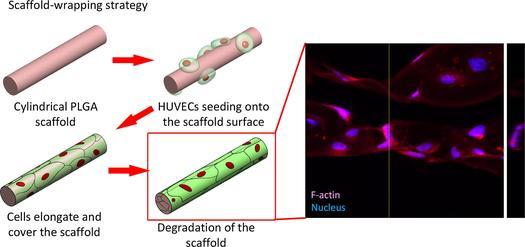

## 1. Introduction

The microvascular system is the most critical and fundamental network in the human body. It not only transports nutrition to peripheral tissues but is also involved in numerous biological processes such as inflammation, nutrition/drug absorption, and cancer metastasis. In tissue engineering the need to supply oxygen and nutrients limits the potential thickness of grown tissue to 150–200 μm [1]. Three approaches have been proposed for the *in vivo* development of vascular lumen: vacuole formation and coalescence, wrapping around extracellular space, and cell death and phagocytosis [2]. In an *in vitro* environment, several approaches have been used in the attempt to develop microvascular networks, including decellularized tissue [3], a biomicrofluid system [4,5], an extracellular matrix gel [6,7], and a biodegradable framework [8]. Although self-orientation and autotubulization characteristics of endothelial cells could be utilized to generate three-dimensional (3D) vascular networks such as the *de novo* ways by co-culturing endothelial cells with other types of cells [9] in decellularized tissue or an extracellular matrix, the random pattern and varying diameter of vascular networks lead to difficulties in scientific observation and quantification. By using soft-lithography techniques, a microvascular network with a desired design can be produced [10,11]. However, the rectangular channels used were dissimilar to *in vivo* vessels and led to difficulties when monitoring the cell condition. Developing a method for generating a microvascular network with a specific pattern in a more biomimetic way not only benefits the *in vitro* development of functional tissues of considerable size but also makes the study of biological response *in vitro* easier and more accurate.

Poly(lactide-co-glycolide) (PLGA) is a biodegradable and biocompatible material [12,13] approved by the US Food and Drug Administration (FDA). It is produced by the polymerization of a lactic acid and glycolic acid solution, with properties that depend on the ratio of the two acids. PLGA degrades to lactic and glycolic acids via hydrolysis [14]. Due to its good mechanical strength and biodegradable/biocompatible properties, PLGA can be electrospun into fibers to create a porous scaffold for use in tissue engineering in applications such as bone tissue [15], cardiac tissue [16], intestinal stents [17], and skin regeneration [18]. It has also been widely used as a material in tissue engineering scaffolds [19] or nanovehicles for drug transport and release [20]. For microvessel development, Pirlo et al. [21] dissolved sodium chloride in a PLGA solution and used a polydimethylsiloxane (PDMS) mold to cast porous rectangular biopaper for use as a scaffold. They then produced a 3D microvascular network by seeding human umbilical vein endothelial cells (HUVECs) on the paper and stacking them layer by layer. The relatively high mechanical strength of PLGA allows it to be processed by femtosecond laser ablation into a 50-μm-thick solid microvascular scaffold made of solid branches 50 μm in diameter [22]. A nanostructured pattern can also be placed directly on a channel surface by casting PLGA on an anodic-aluminum modified hemi-cylindrical template to generate a microvessel scaffold with nanopatterned inner walls [23]. These features make PLGA a popular material in the field of tissue engineering and regenerative medicine.

To develop a microvascular network *in vitro*, a biocompatible extracellular matrix and soft lithography are typically integrated to fabricate a template for culturing endothelial cells [24]. Zheng et al. [25] fabricated a collagen-based channel network by pressing collagen gel on a patterned PDMS stamp and then combined the patterned gel with a flat gel to generate a network structure of 100-μm-thick fibers. They then seeded HUVECs inside the channels to develop a vascular chip, which expressed endothelial markers CD31 and vascular endothelial cadherin (VE-cadherin), and used it to study angiogenesis and thrombosis. With assistance from a live-cell imaging system, Wong and Searson [26] developed a cylindrical microvascular chip (diameter 150 μm) capable of expressing endothelial marker CD31 for studying the interaction between breast cancer and endothelial cells, as well as the intravasation movement of cancer cells. Kim et al. [27] developed a functional microvascular chip from matrix gel by designing five parallel grooves and utilizing the autotubulization ability of endothelial cells to randomly grow HUVECs inside the central channel. The authors were then able to investigate the expression of vascular endothelial markers and interactions among endothelial cells with pericytes, cancer cells, and leukocytes. Wang et al. [28] fabricated a network with circular channels to mimic the real physiological structure of capillary networks. However, most of the methods described above were based on rectangular tubes and require a circulation pump to maintain a regular influx of fresh medium into the channels, which increases the risk of contamination. In addition, the real diameter of microvessels ranges from a few to a hundred micrometers [29], and only very few methods can work on microvascular networks containing microchannels with diameters less than 50 μm. This limitation may be due to the difficulty of aligning two microvascular templates within a narrow window. Although randomly grown endothelial cells can form microvascular networks with small diameters, the lack of a uniform structure hinders some areas of biophysical research, such as the fluid mechanics of the blood stream. Furthermore, some biodegradable materials become milky and opaque after being immersed in a culture medium for less than 24 h, making monitoring of the condition of the cultured cells inside the channels challenging.

**Scheme 1.**
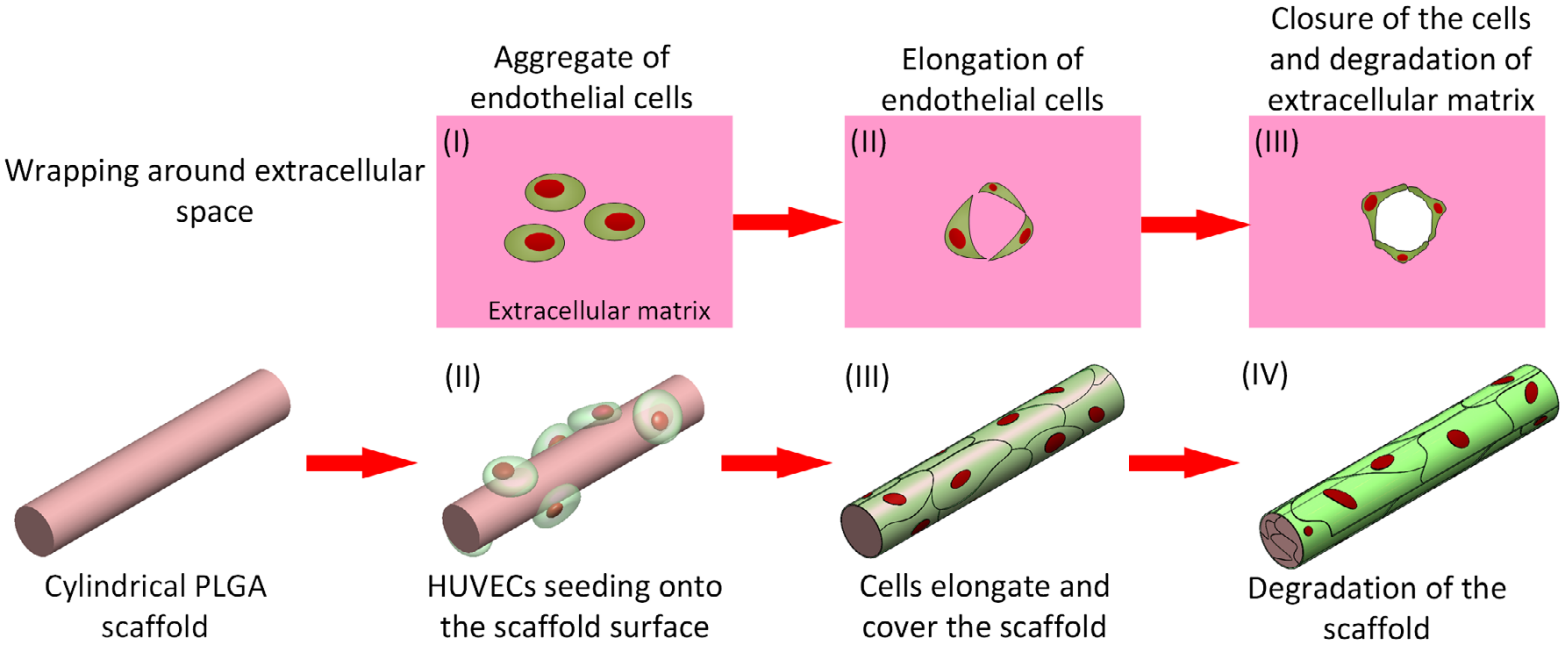
Formation of a microvessel through the scaffold-wrapping strategy.

By mimicking the elongating and wrapping approach of vasculogenesis, we propose a scaffold-wrapping strategy for developing a microvascular network with desired pattern (Scheme 1). Using PLGA as the structural material, a less than 50-μm-diameter cylindrical scaffold with a specified pattern is generated and reproduced via soft lithography. After seeding and growing endothelial cells on our scaffolds, the expressions of two important endothelial markers, CD31 and VE-cadherin, are tested for evaluating cell performances. Our approach not only provides an easier way of culturing/observing early stages of vascular development *in vitro*, but is also an inspiring idea for generating artificial microvessels in a more physiological way.

## 2. Materials and methods

### 2.1. PDMS replica mold preparation

**Scheme 2.**
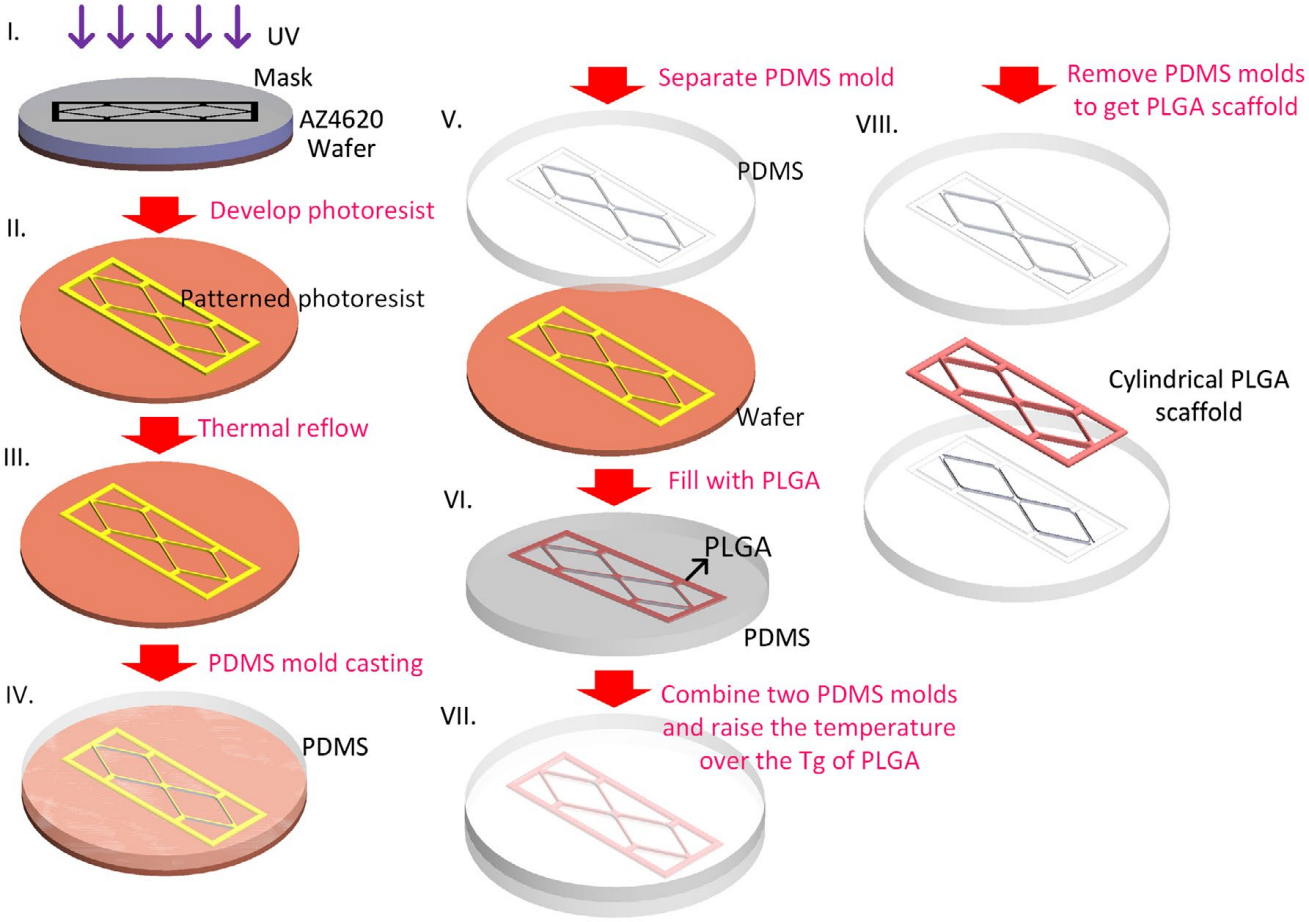
Schematic of the cylindrical PLGA scaffold fabrication. Tg stands for glass transition temperature.

A soft-lithography process was used to make the PDMS (Dow Corning® Sylgard 184; Dow Corning, Midland, MI, USA) replica mold (Scheme 2). Photoresist AZ4620 (Merck, Darmstadt, Germany) was spin-coated on a silicon wafer to generate a 13-μm-thick film. After soft baking (65°C for 1 min, followed by 2 min at 110 C and 1 min at 65°C), the photoresist was exposed to 400 mJ cm^–2^ using a single-side mask aligner (OAI-500). After developing, the wafer was heated to 110°C for 15 min to thermally reflow the cuboid photoresist into a hemi-cylinder. A PDMS solution (base: curing agent; 1:10) was then poured on the wafer surface with its network of hemi-cylinders and cured overnight in a vacuum oven at 50°C to obtain a replica mold of the PDMS.

### 2.2. 3,6-bis(1-methyl-4-vinylpyridium) carbazole diiodide (BMVC) production

BMVC made from 3,6-dibromocarbazole was used for scaffold labeling, following a previously reported procedure [30].

### 2.3. Microvascular scaffold fabrication

To produce the microvascular scaffold, we made a PLGA 50/50 (inherent viscosity (0.2% in chloroform, 25°C) = 0.52 dl g^–1^; Lot: P0406511002, Green Square Material Inc., Taoyuan, Taiwan) solution by dissolving PLGA powder into acetone in a 1:7 w/w ratio. To fabricate the BMVC containing scaffold BMVC solution was mixed with PLGA solution at a dilution of 1:1000. The PLGA solution was spread on the PDMS replica mold and placed overnight at room temperature to allow the acetone solvent to evaporate. By joining and carefully aligning two hemi-cylinders and incubating them at 60°C (which is higher than the glass transition temperature of PLGA) for 1 h, we were able to obtain a microvascular scaffold with cylindrical branches (Scheme 2).

### 2.4. WST-1 assay

To examine HUVECs viability during culturing on our PLGA scaffold, a ready-to-use cell proliferation reagent WST-1 (Cat: K304, BioVision, Milpitas, CA, USA) was used. PLGA 50/50 powder was dissolved in acetone in a 1:7 w/w ratio and gently separated onto the flat PDMS mold to make flat PLGA membranes. The flat PLGA membranes were then cut into small circles which fitted the area of the 96 wells and rinsed with 70% alcohol followed by exposed under UV light overnight. Those circular PLGA membranes were then stuck on the bottom of a 96-well dish and coated with 0.5% gelatin. 1500 HUVECs were seeded in each well and WST-1 was used for measuring the increment of cell numbers after culturing for three and seven days.

### 2.5. Cell culture

Primary HUVECs (Lot: H-UV001, BCRC, Taiwan) were maintained in a 0.5% gelatin (Cat: G1890, Sigma-Aldrich, St. Louis, MO, USA) coated culture dish using M200 medium (Cat: M200500, Thermo Fisher Scientific, Waltham, MA, USA) with the addition of a low serum growth supplement (LSGS) (Cat: S00310, Thermo Fisher Scientific). The PLGA scaffolds were sterilized by rinsing with 70% EtOH and exposed overnight to a UV lamp (the BMVC doped scaffold did not go through this UV exposed procedure). After sterilization, the PLGA scaffolds were coated with 0.5% gelatin to enhance cell attachment. We used a two side seeding strategy in seeding the HUVECs on the scaffold surface. One ml of culture medium with a trypsinized HUVEC concentration of 1.3 × 10^5^ cm^3^ was poured on the surface of a 3 × 3 cm^2^ PLGA scaffold. After incubation for 1 h at 37°C, the PLGA scaffold was flipped over and cells at the same concentration were poured on the other side of the PLGA scaffold and incubated at 37°C for another 1 h. The HUVEC-seeded scaffold was then transferred to a new culture dish and cultured at 37°C in a humidified CO_2_ incubator. Scaffolds cultured for three days were used on confocal microscopy observation, and scaffolds cultured for one week were used for traditional fluorescence microscopy analysis.

### 2.6. Fluorescent staining

The PLGA scaffold was fixed in 4% paraformaldehyde for 15 min followed by treated with 0.1% Triton X-100 (Cat: T9284, Sigma-Aldrich) for 10 min, and then blocked with 5% goat serum for 30 min. The actin filament was stained with Alexa Fluor 568 phalloidin (Cat: A12380, Thermo Fisher Scientific) for 30 min and the nucleus was stained with DAPI (Cat: 32670, Sigma-Aldrich) for 10 min. For the triple staining of the vascular endothelial cell marker CD31 and VE-cadherin, samples were incubated in a polyclonal rabbit-anti CD31 antibody with a dilution of 1:300 (Cat: bs-0195R, Bioss, Boston, MA, USA) overnight at 4°C, followed by incubation with a secondary goat anti-rabbit Alexa Fluor 568 antibody. The samples were then incubated with a mouse-anti CD144 antibody with a dilution of 1:1000 (Cat: 16B1, eBioscience, San Diego, CA, USA) for 2.5 h at room temperature, followed by incubation with a secondary goat anti-mouse Alexa Fluor 488 antibody. DAPI was then used to stain the nuclei.

## 3. Results and discussion

### 3.1. Preparation of PDMS mold

**Figure 1.**
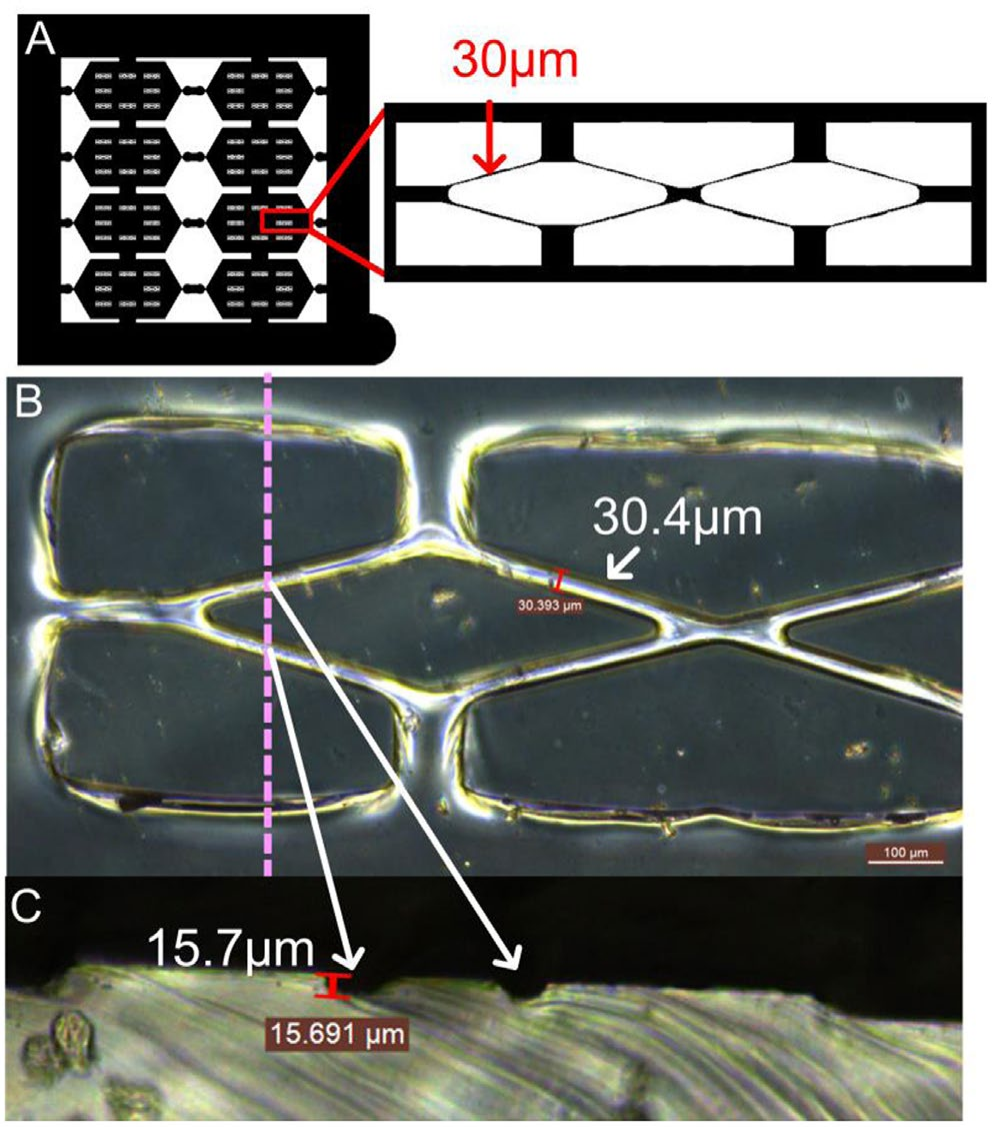
Mask design and PDMS replica mold. (A) Pattern of the mask which contained several double rhombuses with 30-μm-wide edges. (B) Front view of the PDMS replica mold. (C) Cross section of the PDMS mold cut at the dotted line to reveal the hemi-cylindrical structure.

Fabricating a desired cylindrical scaffold with an average tube diameter around 30 μm began with mask design, which was shown in Figure 1(A), a 4 × 4 cm outer and a 3 × 3 cm inner square were included. The inner square was divided into eight individual hexagons, each of which contained eight double rhombuses with a width of about 30 μm. The double rhombus pattern was designed for mimicking a smaller microvascular network with the structure of branch and join-of-branch pattern. PDMS is a highly permeable, biocompatible elastic polymer that has been widely used in micromolding and as the microvascular channel for culturing endothelial cells [31]. In order to fabricate the PDMS molds with a hemi-cylindrical pattern, a proper photoresist that has a suitable film thickness and thermal reflow properties is preferred. AZ4620 photoresist, which can generate films with thicknesses from 6 to 17 μm and has a thermal reflow temperature of 110°C, was chosen for producing the hemi-cylindrical template. PDMS molds were generated by pouring this polymer onto a thermal reflow treated AZ4620 pattern then cured in a vacuum oven. By cutting the PDMS mold vertically to correspond with the direction of the dotted line, two semicircular shapes with diameters of approximately 13 μm could be observed (Figure 1(B) and (C)). These not only showed the thickness of the photoresist coating but also demonstrated that the photoresist had reflowed desirably. The original photoresist mold was used to reproduce more PDMS molds and retained a high level of accuracy.

### 3.2. Viability of HUVECs on PLGA50/50

**Figure 2.**
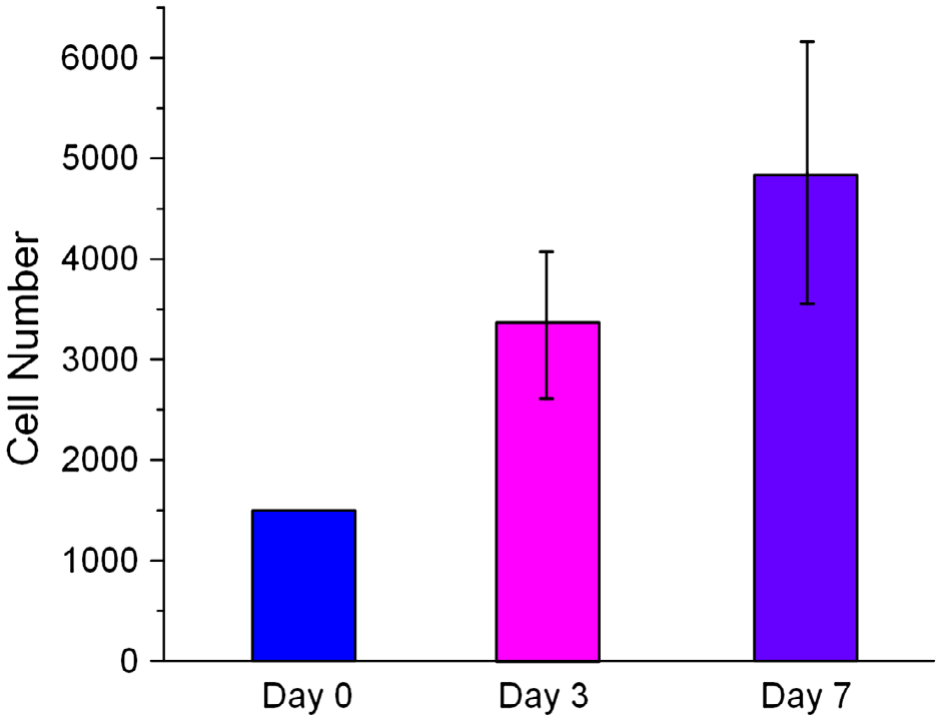
Viability of HUVECs on PLGA50/50 material. Cell numbers were analyzed by WST-1 assay after seeding on PLGA member for three and seven days, respectively (*n* = 5).

Although PLGA was an FDA approved bio-comparable material, we still need to ensure that HUVECs could survive and proliferate on our PLGA scaffold. A WST-1 assay was used to investigate whether the primary HUVECs could proliferate on PLGA50/50 material. The measurement of cell viability was taken at the third and seventh day after seeding. The WST1 result showed that HUVECs doubled its number from day 0 to day 3 and almost doubled its number again from day 3 to day 7 (Figure 2), which indicated that HUVECs could survive on our PLGA material and PLGA50/50 is suitable as a cylindrical scaffold material.

**Figure 3.**
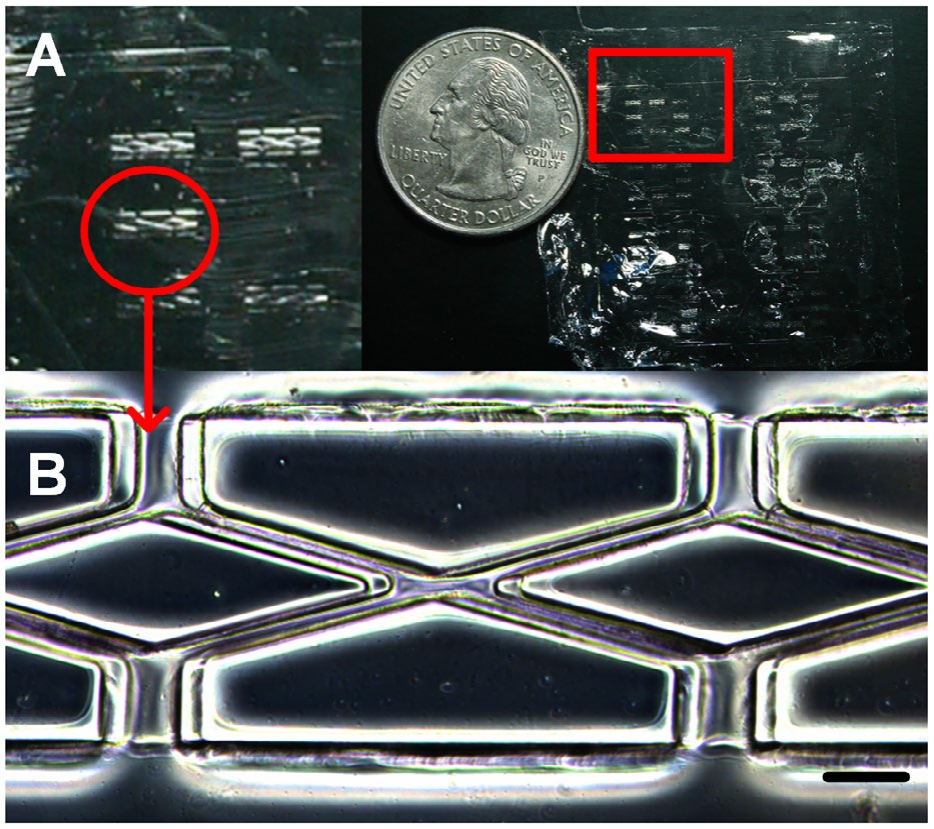
PLGA scaffold. (A) Entire PLGA film with eight individual hexagons (red square). (B) The double rhombus structure within each hexagon. Scale bar: 100 μm.

**Figure 4.**
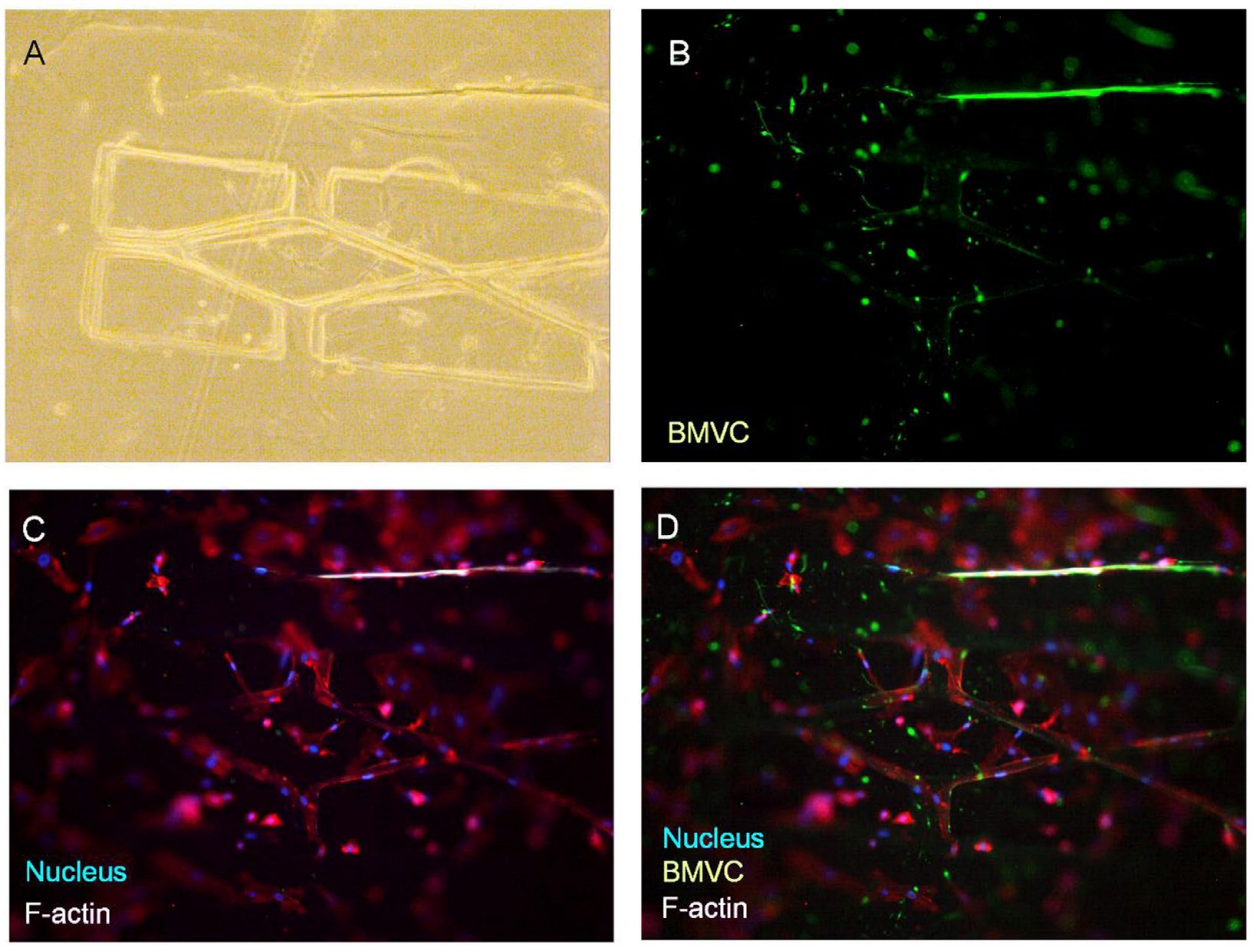
The formation of the microvascular network according to designed pattern. (A) Bright-field image. (B) The fluorescence of the BMVC doped scaffold. (C) Nucleus and actin filaments stained by DAPI and phalloidin. (D) Merged image of the fluorescence from the scaffold, actin, and nucleus. Scale bar: 100 μm.

**Figure 5.**
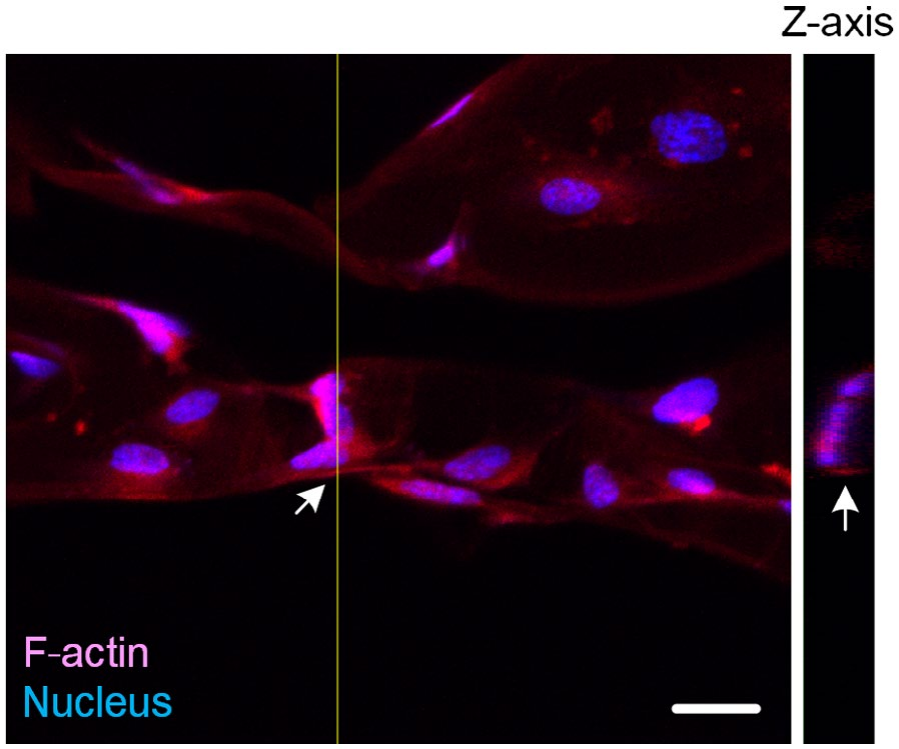
3D confocal microscopy image of HUVE-cultured scaffold. Arrows point out the area in which Z-axis cross-section was performed. Scale bar: 20 μm. Depth of Z-axis: 12 μm.

**Figure 6.**
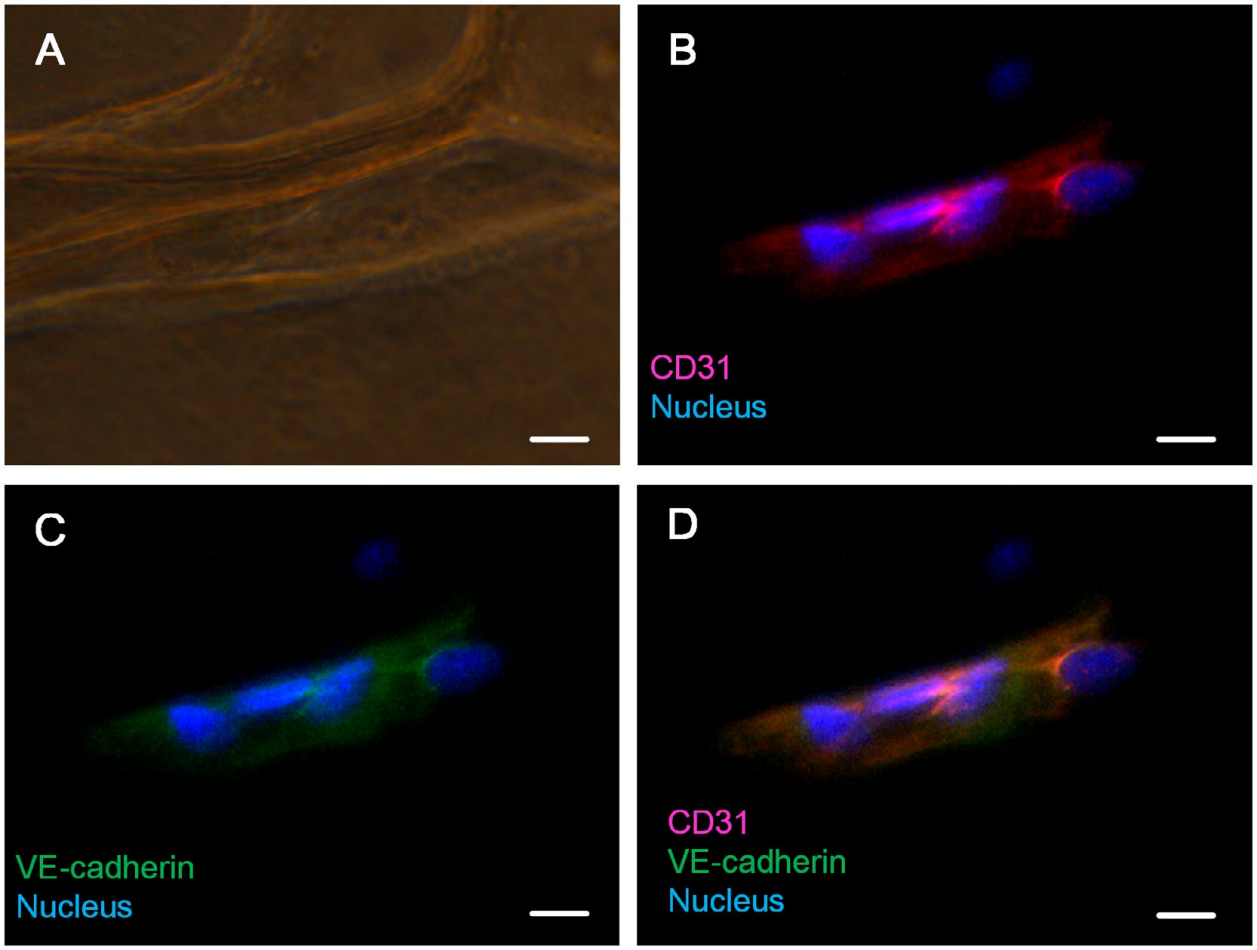
Immunofluorescent staining of CD31 and VE-cadherin. (A) Bright-field image. (B) Merged image of the nucleus and CD31. (C) Merged image of the nucleus and VE-cadherin. (D) Merged image of the nucleus, CD31 and VE-cadherin. Scale bar: 25 μm.

### 3.3. Fabrication of the PLGA scaffold

Since PDMS molds with hemi-cylindrical structure could be produced, we could fabricate cylindrical scaffolds according to the soft-lithography process described in Scheme 2. Due to the high mechanical strength of PLGA, the scaffold could be peeled off from the PDMS mold without destroying the micropattern of the scaffold. Figure 3(A) shows a dry PLGA film with a similar pattern to that of our designed mask. By zooming into the double rhombus section, a clear double rhombus scaffold with a width approximately 29.5 μm could be observed (Figure 3(B)). Some mismatch of the double rhombus structure could be seen in Figure 2, which is due to the difficulty when aligning the two PDMS molds in such a narrow window. The bonding process could be improved in the future by developing a microscope-like device that can monitor the mold through a lens, allowing the two molds to be aligned by adjusting the stage position. The high degree of similarity between the produced scaffold and the PDMS replica mold suggests that our fabrication approach is a feasible method for fabricating cylindrical scaffolds. In this study, a 30-μm-diameter cylinder was generated through soft-lithography; however, it is still relatively large in comparison with the smallest capillary (approximately 3 μm). Changing the photoresist to generate a thinner film for photolithography processing allows the construction of a microvascular scaffold with a smaller diameter. As the thickness of the fabricated scaffold (30 μm) was extremely thin it was very difficult to fit into the holder of our materials testing machine (LRX), hence the physical properties of our dry PLGA film cannot be precisely measured. PLGA has been commonly mixed with other biocompatible materials to generate materials with particular properties, such as calcium phosphate cements for bone regeneration [32], gelatin for neural recovery [33], and collagen with graphene oxide to increase hydrophilicity and thereby stimulate myoblast differentiation [34]. We wanted to combine PLGA with other materials to generate a more hydrophilic and easy-to-handle scaffold with this mold-casting process to fabricate micro-scale scaffolds for future application.

### 3.4. The formation of a microvascular framework

According to our biomimetic scaffold-wrapping strategy, HUVECs were seeded on our cylindrical PLGA scaffolds and then analysis of the growth pattern of the seeded endothelial cells was performed. In a bright-field image (Figure 4(A)), the double-rhombus structure of the cylindrical scaffold could be observed. To distinguish the cylindrical scaffold under traditional fluorescence microscopy observation, we added fluorescent small organic molecule BMVC to the PLGA solution and fabricated a cylindrical scaffold with green fluorescence (Figure 4(B)). By combining the stained actin filament image with that of the stained nucleus, a double-rhombus structure could be seen in the immunofluorescence microscopy image (Figure 4(C)). The growth of HUVECs of our designed pattern was confirmed by merging the fluorescence from the scaffold and fluorescence from the cells (Figure 4(D)). To further evaluate whether HUVECs could grow and wrap the cylindrical scaffold, a 4D confocal microscopy image was performed to analyze the cross-section of the HUVEC-seeded scaffold. A clear hemi-circular shape could be observed on the 3D confocal microscopy image (Figure 5, arrows), which indicated the cylindrical scaffold was covered by HUVECs and will be able to turn into vascular lumen after the degradation of the scaffold. The customizable degradation rate and physical properties that PLGA possesses make it a popular material in tissue engineering [35]. Due to the use of equal proportions of PLA and PGA (PLGA 50/50) for fabricating our microvascular scaffolds they had a shorter degradation half-life of two weeks [36] than that (PLGA 85/15) used in our previous study [23], which resulted in a dramatic drop in mechanical strength after soaking in medium [37]. The decrease in mechanical strength made our cylindrical scaffold brittle and susceptible to wrinkling, which hinders immunofluorescent staining, microscopy observations, and measurements of the mechanical properties of cell-covered scaffolds. Some cells in our fluorescence image were out of focus, probably because the cells were located opposite the plane of observation or because of wrinkles in the scaffold.

### 3.5. Examination of the CD31 and VE-cadherin expression on HUVECs cultured scaffold

To further analyze whether HUVECs exhibit normal physiological characteristics while growing on our cylindrical scaffold, immunostaining of CD31 and VE-cadherin vascular endothelial markers was used. CD31 (also known as PECAM-1) is an adhesion molecule of the immunoglobulin superfamily which is expressed on the surface of palate, hemocyte, leukocyte, and vascular endothelial cells. CD31 not only functions as a regulator protein in lymphocyte transmigration in the immune system but also works as an adherens junction to control the permeability of an endothelial cell and the integrity of the endothelial cell to cell junction [38]. During angiogenesis, CD31 is critical for the migration of endothelial cells, the development of cell to cell junctions, and the maturation of cell to extracellular matrix interactions [39,40]. VE-cadherin is the basic adhesion junction in the endothelial cell, which is important for cell to cell contacts [41]. During early vascular development in mouse embryos, VE-cadherin plays a crucial role in determining the vascular endothelial cell polarity and the formation of vascular lumen [42]. VE-cadherin is also required for maintaining the structural integrity of the blood vessel and preventing the aggregation of vascular endothelial cells during vasculogenesis [43]. By controlling vascular permeability to solutes, VE-cadherin is involved in several complex signaling transduction pathways which strengthen or weaken the endothelial cell–cell connections, controlling vascular permeability and maintaining the homeostasis of the blood [44]. Figure 6(A) is a bright-field image showing the structure of the cylindrical scaffold. Figure 6(B) is a merged image of the CD31 (red) and nucleus (blue). The staining of CD31 suggested that the scaffold-covering HUVECs express its biological marker, whereas the expression of VE-cadherin (Figure 6(C)) demonstrated that these scaffold-covering HUVECs has normal cell-to-cell junctions. The coexpression and colocalization of CD31 and VE-cadherin of the scaffold-covering HUVECs (Figure 6(D)) indicated that our scaffold-wrapping strategy has the potential for developing a microvascular network which functions as an *in vivo* one.

## 4. Conclusions

The development of microvascular networks has been attracting interest in recent years, especially in the field of tissue engineering [45,46]. The need to create artificial microvascular networks requires a better understanding of microvascular biofunctions [47], to not only produce microvascular networks that can better mimic *in vivo* networks but also support biomedical studies. Previously reported microvascular chips have typically been made either by punching a microrod through a biocompatible gel [26] or by fabrication using soft lithography; however, difficulties still remain in developing scaffolds/channels with a cylindrical structure and desired net pattern. In this study, we propose a strategy for generating a microvascular network with diameters of less than 50 μm in a more physiological approach. Unlike previous studies, which usually begin by seeding HUVECs inside the designed channel, we used a scaffold-wrapping method for generating our microvascular network. This scaffold wrapping strategy was inspired by a published *in vivo* vascular lumen formatting method [2] and has advantages for facilitating the cell culture and observational process at the beginning of the developmental stage. Soft lithography was used to fabricate PLGA cylindrical scaffolds and covering HUVECs on both sides of the PLGA scaffold to create a microvascular network to our designed pattern. The expression of vascular endothelial marker CD31 and VE-cadherin in our scaffold-covered HUVECs indicated that our scaffold-wrapping strategy is highly feasible for the development of microvascular network *in vitro*. The direct culture of endothelial cells onto the scaffold also allowed easier observation of cell state. This approach can be used with the traditional culturing method and reduces the need for support from a circulating pump system in the early stages of culturing. The thin and solid structure of the PLGA scaffold allows it to be easily introduced to other vascular cells by seeding and co-culturing them layer by layer or stacking the cell-covered vascular scaffolds together to form the framework for building large tissues. In the future, we plan to encapsulate our cell-covered scaffold into collagen gel and hope cells can attach to the collagen and maintain the shape of the scaffold after the template is gone. An *in vivo* implantation study is also desired to evaluate the potential of our HUVECs-covered cylindrical scaffold in developing into a functional microvessel. The success of our scaffold-wrapping approach provides an insight for *in vitro* engineered tissues/organs in a physiologically relevant way.

## Disclosure statement

No potential conflict of interest was reported by the authors.

## Funding

The authors would like to offer their thanks to the Ministry of Science and Technology of Taiwan for their financial support of this research [grant number MOST-103-2221-E-005-046-MY2].
